# The Status of Assistant Medical Officers in Mental Hospitals

**Published:** 1913-03-08

**Authors:** 


### The Hospital

The Workers' Newspaper of Administrative Medicine and Institutional Life, Administration, National Insurance and Health.

No. 1392, Vol. LIII. SATURDAY, MARCH 8, 1913. PRICE ONE PENNY.

### THE STATUS OF ASSISTANT MEDICAL OFFICERS IN MENTAL HOSPITALS.

In the interests of the public the question of the status of the assistant medical officers in our large hospitals for mental diseases is one which demands immediate attention. Indeed, any possible doubt of its importance must have been completely brushed away for all who had the opportunity of reading the discussion at the quarterly meeting of the Medico-Psychological Association of Great Britain, which we published practically in full last week. This class plays a necessary and increasingly important part in a large public service, while receiving no recognised status in keeping with its work. The burden of administration which falls upon the medical superintendent of such an institution leaves little time for purely medical work. He can in that respect do little more than act as consultant to his junior colleagues. The general medical work must fall upon the assistant medical officer. It is, therefore, essential that the service should attract and retain the best type of medical man. The work is of a highly specialised nature. It is a branch of medicine in itself, and is more and more calling for peculiar qualifications.

This is now so clearly recognised that, for example, the Universities of Cambridge, Edinburgh, Durham, Leeds, and Manchester are instituting a diploma in Psychological Medicine. In the future it is probable that the man who hopes for success in this branch of medicine must obtain this special qualification. This means the expenditure of additional money, time, and energy. Do the present conditions of the service offer such rewards as will induce the stamp of man who is wanted to take up the work and continue in it? The prizes are not many and the blanks are not few. The natural ambition of every young medical man who enters this field is to become a medical superintendent. From the number of such vacancies yearly it is inevitable that disappointment must await a large number, even after many weary years of waiting. There are also many men who, without the administrative capacity necessary for the controlling heads of such institutions, are yet most capable physicians. Perhaps some are of greater value to the hospital by their work as physician than the men with administrative talents. Are the rewards fairly divided? We would not for one moment advocate the lowering of the salaries received by the medical superintendents. Their duties and their responsibilities fully deserve the remuneration which they receive. It is a case for levelling up and not down.

The assistant medical officers have a legitimate grievance. It is probably one of the gravest indictments of the present system under which our great mental hospitals are managed that so many of the junior medical staff are condemned to celibacy. If their grievances are not remedied their dissatisfaction is bound to have a detrimental effect upon the service. With few exceptions, the only chance for such men to make a home for themselves and enjoy a normal home life is to become medical superintendents. This for the great number who are doing valuable work is impossible. There are no cogent administrative reasons why the number of married assistant medical officers might not be largely increased. There are strong reasons why men who are trained and skilled in the treatment of mental disease should be given a recognised status and permitted to live under such conditions as will enable them to make a home for themselves. No reasonable complaint can be made with respect to the payment given to the junior assistants on entering the service. A salary of one hundred and fifty pounds with all found is fair remuneration for a newly qualified man if there be a prospect that by devoting himself to his speciality he can obtain a competence with an opportunity of marriage even if he should not attain to the dignity of a medical superintendent. At present there is not such a reasonable prospect. The conditions of life under which the assistant medical officer works are those to which no man should, after years of service, be forced to adapt himself under penalty of giving up the special work for which he has qualified.

The position of the assistant medical officer has altered and is altering. There is less chance of promotion than there used to be, and the qualifications requisite for the work are higher. His status has not advanced with his responsibilities. Where a staff includes several assistants the privilege of permission to marry might well be extended without interfering with the effective working of the hospital. In the interests of the patients, and therefore of the public, it would be well if the position of the junior medical staff were improved. The man who devotes his life to such a work deserves recognition and a position at least equal to that of the general practitioner. At present he certainly does not receive this; and the present results are liable to affect the service so disastrously that it is of the utmost importance to make clear the general principles which must guide the inevitable reforms. That is why we have chosen the present week to enforce the moral which arises, and so clearly to those who are acquainted with the facts, from the detailed discussion which we printed last week. Dr. McDowall, Dr. J. Beveridge Spence, Dr. Sargeant each forcibly pressed one or more aspects of the question. But their very just criticisms need to be focussed, like a burning-glass, on the subject of inquiry. That is what The Hospital is determined to do. Reform would have come sooner had the separate criticisms been united in a channel which comprises all aspects of the mental hospital problem, and we are glad to take the present opportunity of reminding all assistant medical officers in mental hospitals throughout the country that the remedy for the system under which they suffer is in their own hands. In the institution they are necessarily separate and often inevitably silent entities. In our columns the many individual voices can all find expression. For it will only be by uniting on a common public platform to make their just grievances known that the assistant medical officer will shake himself free of the shackles of the present system.

